# Identification of hub genes and pathophysiological mechanism related to acute unilateral vestibulopathy by integrated bioinformatics analysis

**DOI:** 10.3389/fneur.2022.987076

**Published:** 2022-09-27

**Authors:** Yajing Cheng, Jianrong Zheng, Ying Zhan, Cong Liu, Bihua Lu, Jun Hu

**Affiliations:** Department of Neurology, Peking University Shenzhen Hospital, Shenzhen, China

**Keywords:** acute unilateral vestibulopathy, inflammation, immune response, bioinformatics analysis, hub genes

## Abstract

**Background:**

Although many pathological mechanisms and etiological hypotheses of acute unilateral vestibulopathy (AUVP) have been reported, but the actual etiology remains to be elucidated.

**Objective:**

This study was based on comprehensive bioinformatics to identify the critical genes of AUVP and explore its pathological mechanism.

**Methods:**

Gene expression profiles of AUVP and normal samples were collected from GSE146230 datasets of the Gene Expression Omnibus (GEO) database. Weighted gene co-expression network analysis (WGCNA) was constructed, and the WGCNA R-package extracted significant modules. The limma R-package was applied to identify differentially expressed genes (DEGs). The common genes of practical modules and DEGs were screened for GO and KEGG pathways analysis. The protein–protein interaction (PPI) layout and hub genes validation was created by Cytoscape software using the link from the STRING database. The functions of hub genes were predicted through the CTD (comparative genetics database).

**Results:**

A total of 332 common genes were screened from practical modules and DEGs. Functional enrichment analysis revealed that these genes were predominantly associated with inflammation and infection. After construction of PPI, expressions of hub genes, and drawing ROC curves, LILRB2, FPR1, AQP9, and LILRA1 are highly expressed in AUVP (*p* < 0.05) and have a certain diagnostic efficacy for AUVP (AUC > 0.7), so they were selected as hub genes. The functions of hub genes suggested that the occurrence of AUVP may be related to inflammation, necrosis, hepatomegaly, and other conditions in CTD.

**Conclusion:**

LILRB2, FPR1, AQP9, and LILRA1 may play essential roles in developing AUVP, providing new ideas for diagnosing and treating AUVP.

## Introduction

Acute unilateral vestibulopathy (AUVP), also known as vestibular neuritis, is an acute peripheral vestibular syndrome characterized by acute unilateral loss of peripheral vestibular function without sensitive central nervous system or acute audiological symptoms or signs (1). It is the third most common peripheral vestibular disease after benign paroxysmal positional vertigo (BPPV) and Meniere's disease (2). There were no unified diagnostic criteria for AUVP, so there is no compelling new epidemiological study (3). The onset age of AUVP is usually 30–60 years old (1, 4, 5), and the distribution peak is 40–50 years old (1, 4). It is reported that the annual incidence rate of AUVP is 3.5–15.5 per 100,000 people (1, 4), the recurrence rate is 1.9% (6)−10.7% (7), and approximately 4–9.8% of adult patients and 3.3% of children are treated for acute unilateral vestibular loss (8). Although it is thought to be caused by viral inflammation or potential viral reactivation in vestibular nerve ganglia, the exact cause of vestibular neuritis is unclear (2). Therefore, the treatment methods for AUVP are various, such as corticosteroids, antiviral drugs, and vestibular rehabilitation training (2), but the treatment effects were not satisfactory.

The Committee for the Classification of Vestibular Disorders of the Bárány Society divides the diagnostic criteria of AUVP into four categories: 1. “Acute Unilateral Vestibulopathy,” 2. “Acute Unilateral Vestibulopathy in Evolution,” 3. “Probable Acute Unilateral Vestibulopathy” and 4. “History of Acute Unilateral Vestibulopathy” (1). The diagnosis of AUVP is based on the patient's medical history, bedside examination, and laboratory evaluation. It is worth noting that since there is no precise detection method for AUVP, its diagnosis needs to exclude central lesions and various other peripheral vestibular disorders. In addition, a pathological examination is a gold standard for diagnosis, but it is not easy to implement clinically. Thus, there is still a lack of ideal indicators in clinical practice. The viral hypothesis of AUVP that Bell's palsy and some types of acute hearing loss are likely to be related to viral infection, has not been confirmed so far (9–12). It has been reported that an autopsy of patients with AUVP showed neuroinflammatory vestibular degeneration (13). It is speculated that HSV-1, as a concurrent factor damaging the immune system, replicates and induces inflammation and edema, resulting in secondary cell damage to vestibular ganglion cells and axons in bone canals, which may also explain the therapeutic effect of steroids in the acute phase of AUVP (1). However, these findings have not confirmed the relationship between inflammation and virus hypothesis and AUVP. Therefore, improving understanding of the molecular mechanisms of AUVP is necessary to predict prognosis and develop therapeutic strategies targeting target genes.

With the rapid development and application of the gene chip and sequencing technology, NCBI (National Center for Biotechnology Information) established the GEO (Gene Expression Omnibus, https://www.ncbi.nlm.nih.gov/geo/) database in 2000 and maintained it. The GEO database collates high-throughput genomic data uploaded by researchers around the world. It is an international repository that archives and freely distributes high-throughput gene expression data (14). Through this database, researchers can easily download all kinds of sequencing data to study tumors, cerebrovascular diseases, neurodegenerative diseases, basic molecular biology, cardiovascular diseases, neuro-otology, and other fields (15–17). At present, research on AUVP has been reported and the original sequencing data have been uploaded (18). Therefore, using an online platform to integrate and analyze the existing high-throughput data can help to mine new biological targets, and provide big data support for follow-up experimental research. WGCNA is a commonly used high-throughput data analysis method to mine module information by analyzing the similarity of gene expression (19). Compared with previous clustering methods, WGCNA clustering has more biological significance. It not only focuses on differentially expressed genes (DEGs) but also makes full use of data information to transform thousands of genes and traits into several gene modules associated with clinical traits, thus eliminating the problem of multiple hypothesis test correction, and the results are more reliable.

This study aims to screen for critical biological processes and essential genes related to the development of AUVP based on a comprehensive bioinformatics analysis. We downloaded the gene expression profiles of AUVP and normal blood samples. After determining the common genes of significant modules and differentially expressed genes (DEGs) between diseased samples and healthy subjects, a cluster analysis and a functional enrichment analysis of common genes were carried out to explore the biological pathways in the processes of AUVP. In addition, the protein-protein interaction (PPI) network of common genes was constructed to find the potentially critical genes in developing AUVP. The hub genes were identified by drawing ROC curves. Finally, the functions of hub genes were predicted through the Comparative Toxicogenomics Database (CTD). The identified hub genes may play an essential role in developing AUVP. These findings provide a reference for exploring the pathological mechanism of AUVP.

## Materials and methods

### Data collection and data preprocessing

The keywords “Acute Unilateral Vestibulopathy,” “vertigo,” and “Vestibulopathy” were searched through the GEO database. Finally, the data set related to AUVP was selected as GSE146230 (18), including 10 control group samples and 10 AUVP patient samples. Before analyzing the dataset, we need to screen the gene probe in the dataset. First, if the gene probe does not have a corresponding gene, the expression of this gene probe will be excluded; if one gene probe corresponds to multiple genes, the face of this gene probe will be banned. If one gene has two or more gene probes, the average expression of all the probes corresponding to the gene in each sample will be retained (20). As a result, 18,782 genes were included and will be further analyzed.

### Gene co-expression network construction and identification of key modules

When constructing the weighted gene co-expression network, the “SD” function in R software was used to calculate the standard deviation of each gene and arrange it in descending order. Genes with the top 5,000 highest standard deviations were obtained for further analysis. Pearson's correlation coefficient among 5,000 genes was calculated to measure the degree of co-expression among genes. The network topology was analyzed through the “picksoftthreshold” function in the WGCNA package of R software. A hierarchical clustering tree of network modules was performed, and the number of modules was determined using the “cutreeDynamic” function with a minimum size module of 30 genes. Finally, we used the Dynamic Tree Cut approach to merge highly correlated modules using a height cut of 0.25 (21). Co-expression modules are represented by module colors, noted in the first row of the horizontal color bar. The correlation coefficients cor and *P-*values of gene modules and groups were calculated according to Pearson's correlation and visualized in the form of heatmap through the “labeledheatmap” function. The smaller the *p*-value and the larger the cor, the stronger correlation between AUVP and the gene module is, and the gene module was selected as the key module.

### Identification of DEGs

First, we used the principal component analysis (PCA) to verify the reliable data and eliminate the system error for further analysis (22). The DEGs between patients with AUVP and control samples were screened by the R language “Limma” package. The selection criteria of DEGs was: | log2FC | > 0.3, and *p* (*T*-test, Empirical Bayes methods) < 0.05 (23, 24). Fold Change (FC) represents the considerable differences between DEGs. The “ggplot2” package of R language was used to visualize the DEGs.

### Screening of candidate genes in AUVP

An intersection of genes in the essential module and DEGs would be obtained by the “ggplot2” package, and the acquired genes were the crucial genes of AUVP.

### Functional enrichment analysis

A functional enrichment analysis can divide hundreds of genes into different pathways and reduce the complexity of the analysis. The KEGG pathway analysis and the GO enrichment analysis of upregulated genes and downregulated genes in DEGs, modules, and common genes were carried out by using “cluster Profiler” of the R language, respectively (23). The GO enrichment analysis covered three aspects of biology namely Biological Process (BP), Cellular Component (CC), and Molecular Function (MF). In this study, *p* < 0.05 was selected as the screening condition, and the top pathways were chosen to explore the biological signal pathways and bodily functions of genes.

### Construction of PPI

The STRING (25) (STRING, http://string.embl.de/) database aims to collect, score, and integrate all publicly available sources of protein-protein interaction information, and complement these with computational predictions. PPI is an essential component of the biological network, which plays a vital role in cell fate determination, signal transduction, and other life processes. It is also a vital link to the occurrence and development of disease. A total of 332 DEGs were introduced into the STRING database to construct the PPI network of key genes in AUVP. The PPI network was analyzed and visualized by Cytoscape software (http://www.cytoscape.org/), and the CytoHubba plug-in analyzed the genes in the PPI network.

### Screening of hub genes

According to the four scoring algorithms, including the maximum neighborhood component (MNC), the density of maximum neighborhood component (DMNC), the maximal clique centrality (MCC), and the Degree of the CytoHubba plug-in, each gene in the PPI network is scored. The genes were ranked according to the level of each score (23). The top 20 genes were selected in each algorithm, and the shared genes of the four scoring algorithms were identified as hub genes.

### Gene expression values of the hub genes in AUVP

To evaluate the clinical value of four hub genes, we used one independent GEO cohort as a validation dataset. The *T*-test analyzed the differences in expression patterns of four hub genes between patients with AUVP and normal samples, and the violin plot using the ggplot2 (V3.3.1) package in the R (V4.0.0) was used for visualization.

### Receiver operating characteristic curve analysis of hub genes

The “pROC” and “ggplot2” packages of R language were used to analyze and visualize the hub gene's ROC curve and compare the hub gene's diagnostic efficiency in patients with AUVP (26). The area value (AUC) under the ROC curve is 0.5–1. The closer the AUC is to 1, the better the diagnostic effect. AUC has lower accuracy when 0.5–0.7, AUC has certain accuracy when 0.7–0.9, and AUC has higher accuracy when it is above 0.9.

### Exploring the common functions of hub genes in the comparative toxicogenomics database

CTD, a robust and publicly available database, was used to find relevant disease associations. To explore the biological function of hub genes, the role of hub genes was predicted by using the CTD from two aspects of chemicals and diseases related to hub genes (27).

### Statistical analyses

WGCNA (version 1.69) and limma (version 1.9.6) was running in R (version 4.0.2) with the default statistics parameter and cut-off values specified in each section. *p* < 0.05 was defined as statistically significant.

## Results

**Figure 1:**
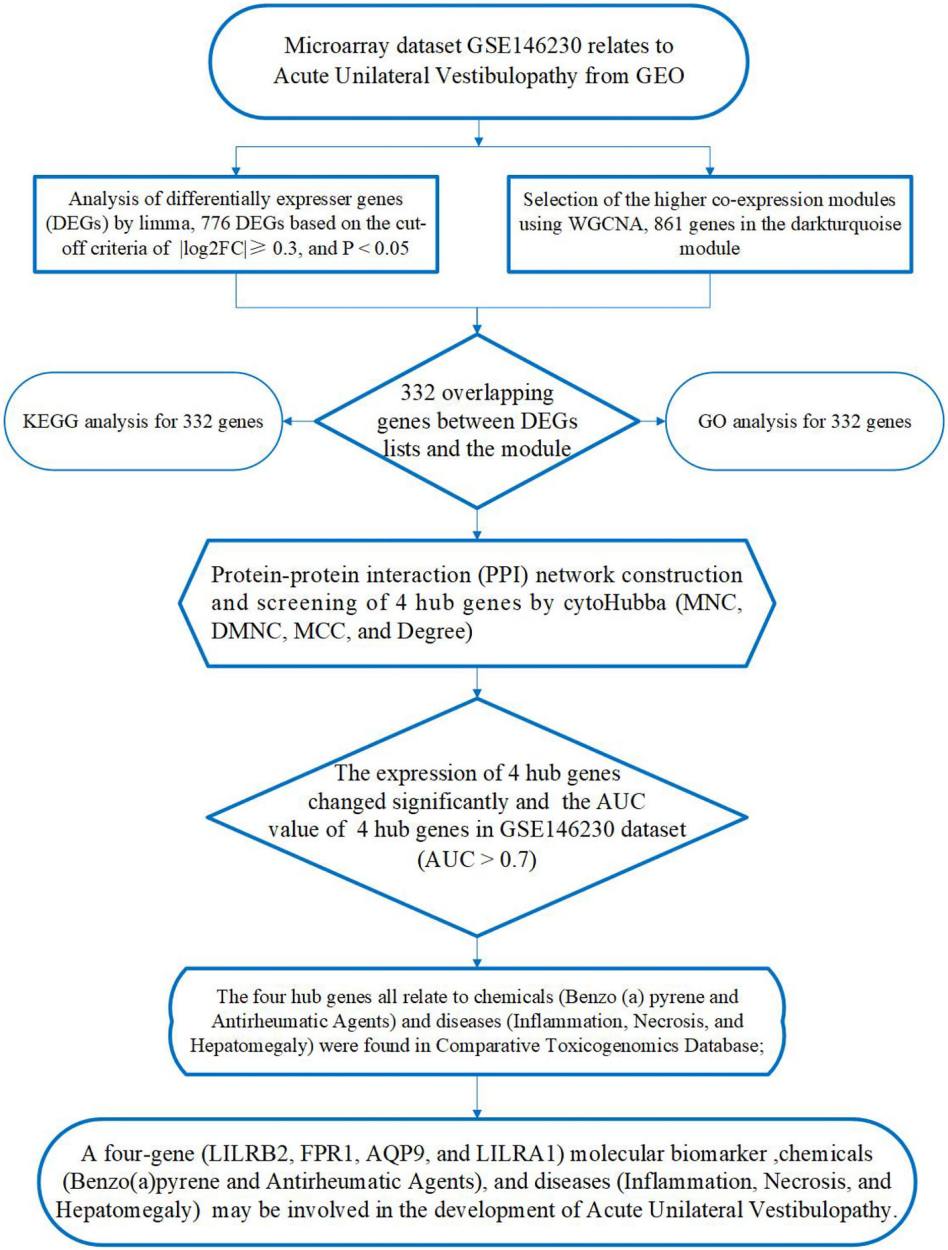
A flowchart presenting a multistep integrated bioinformatics analysis of this study.

Figure 1 presents a multistep integrated bioinformatics analysis of this study.

### Weighted co-expression network construction and key module identification

**Figure 2:**
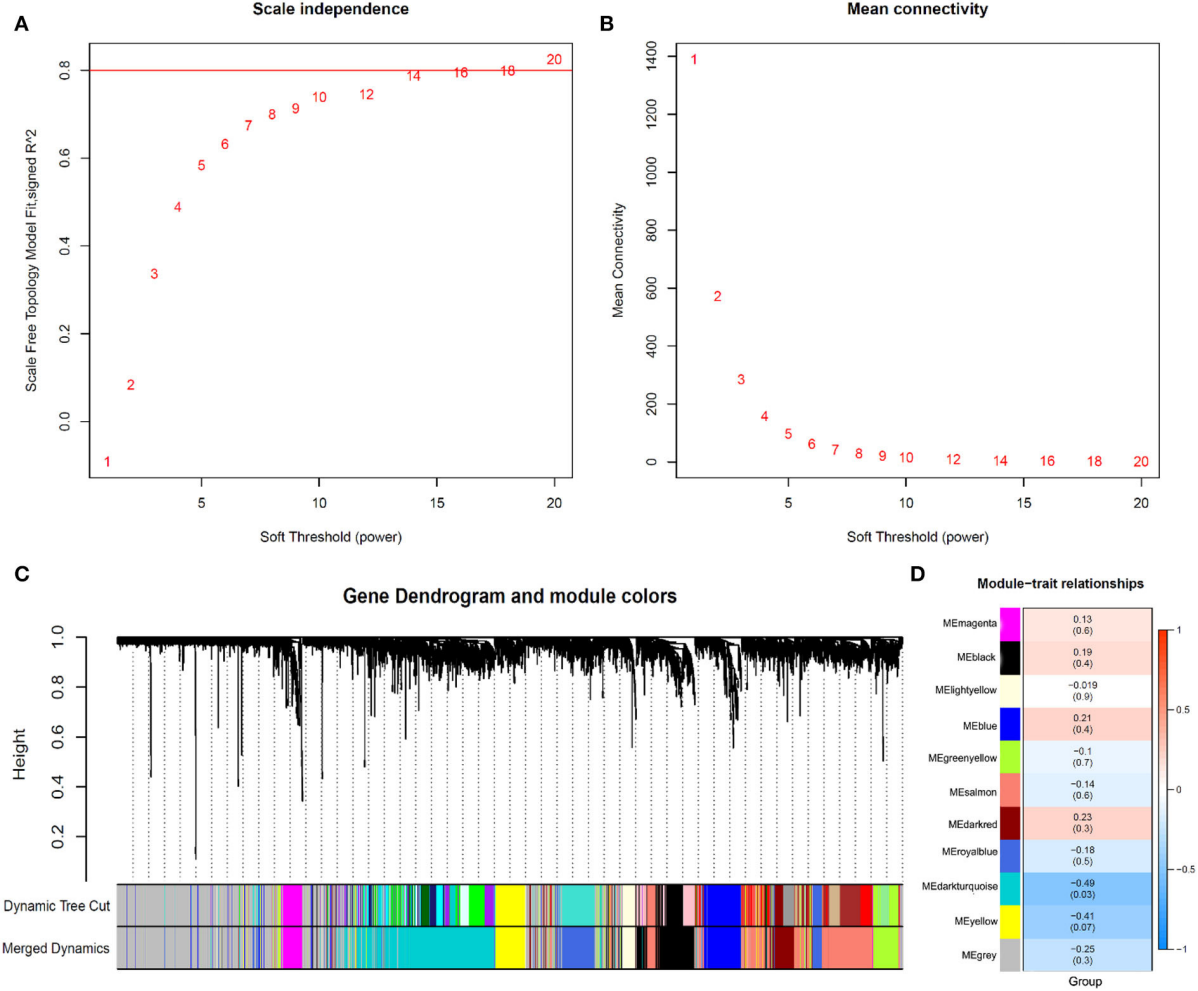
Identification of modules associated with the clinical subtypes of AUVP. **(A)** Analysis of the scale-free fit index for different soft-thresholding power. **(B)** Analysis of the mean connectivity for different soft-thresholding power. **(C)** Dendrogram of all differentially expressed genes clustered. **(D)** Heatmap of the correlation between gene modules and clinical information of AUVP.

The β (β = 20, R2 = 0.89) value was selected to ensure the constructed scale-free co-expression network (Figures 2A,B). After successfully constructing the scale-free co-expression network, using the algorithm recognition module of the dynamic cut tree, the gene expression value in the module is very similar. After the highly similar modules were merged, a total of 11 co-expression modules were identified, while the gray module retained the genes that were not co-expressed (Figure 2C). After the module was cut, the grouping was combined with each module, and the heat map of the correlation between the module and the group was calculated using the “labeled heatmaps” function of the WGCNA package. The results showed that the correlation between the MEdarkturquoise module and patients with AUVP was the greatest, and the *p*-value was the smallest, so there was a significant correlation between the MEdarkturquoise module and patients with AUVP (Figure 2D). The 861 genes in the MEdarkturquoise module will be analyzed.

### Identification of DEGs

**Figure 3:**
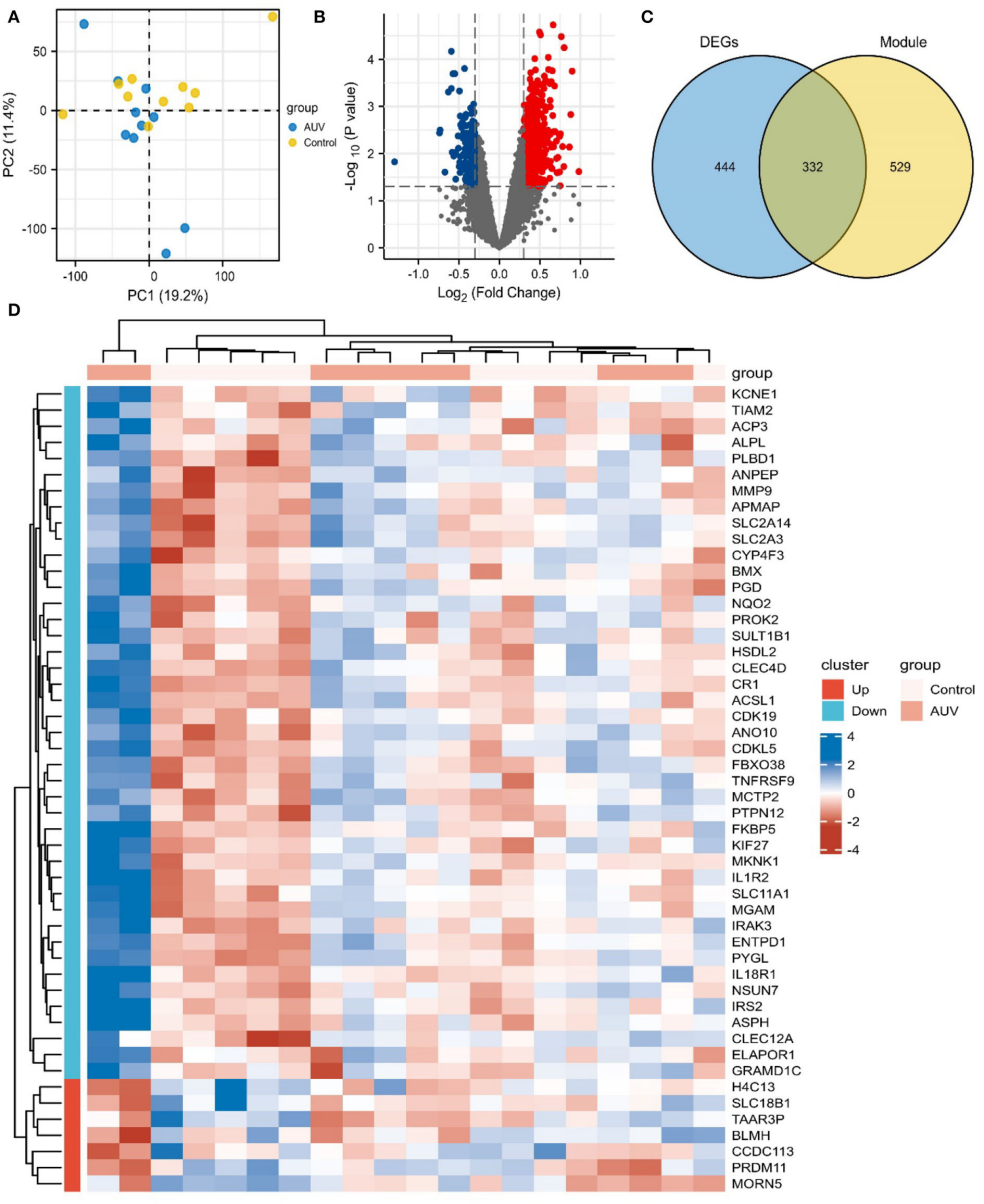
Identification of differentially expressed genes (DEGs) and Key genes of AUVP. **(A)** The principal component analysis biplot of the gene expression profiler between patients with AUVP and control samples. **(B)** Volcano plot of DEGs in AUVP samples. Red: upregulated; Blue: downregulated; Gray: normal. **(C)** The Venn plot of 332 common genes in the MEdarkturquoise module and DEGs. **(D)** The Top 50 DEGs of AUVP.

In this study, the quality control (QC) of data was assessed by the principal component analysis (PCA) (Figure 3A). The DEGs between patients with AUVP and control samples were screened by the R language “limma” package. The selection criteria of DEGs are: | log2FC | > 0.3, *p* < 0.05. As shown in Figure 3B, 776 differential genes were obtained, including 615 upregulated and 161 downregulated genes, red indicates upregulated genes, blue indicates downregulated genes, and gray indicates non-differential genes.

The “ggplot2” package of R software was used to intersect the genes in the key modules and DEGs, and 332 key genes of AUVP were obtained (Figure 3C). The “ComplexHeatmap” package of R language was used to visualize the expression level of the Top 50 DEGs (Figure 3D).

### Functional enrichment analysis

**Figure 4:**
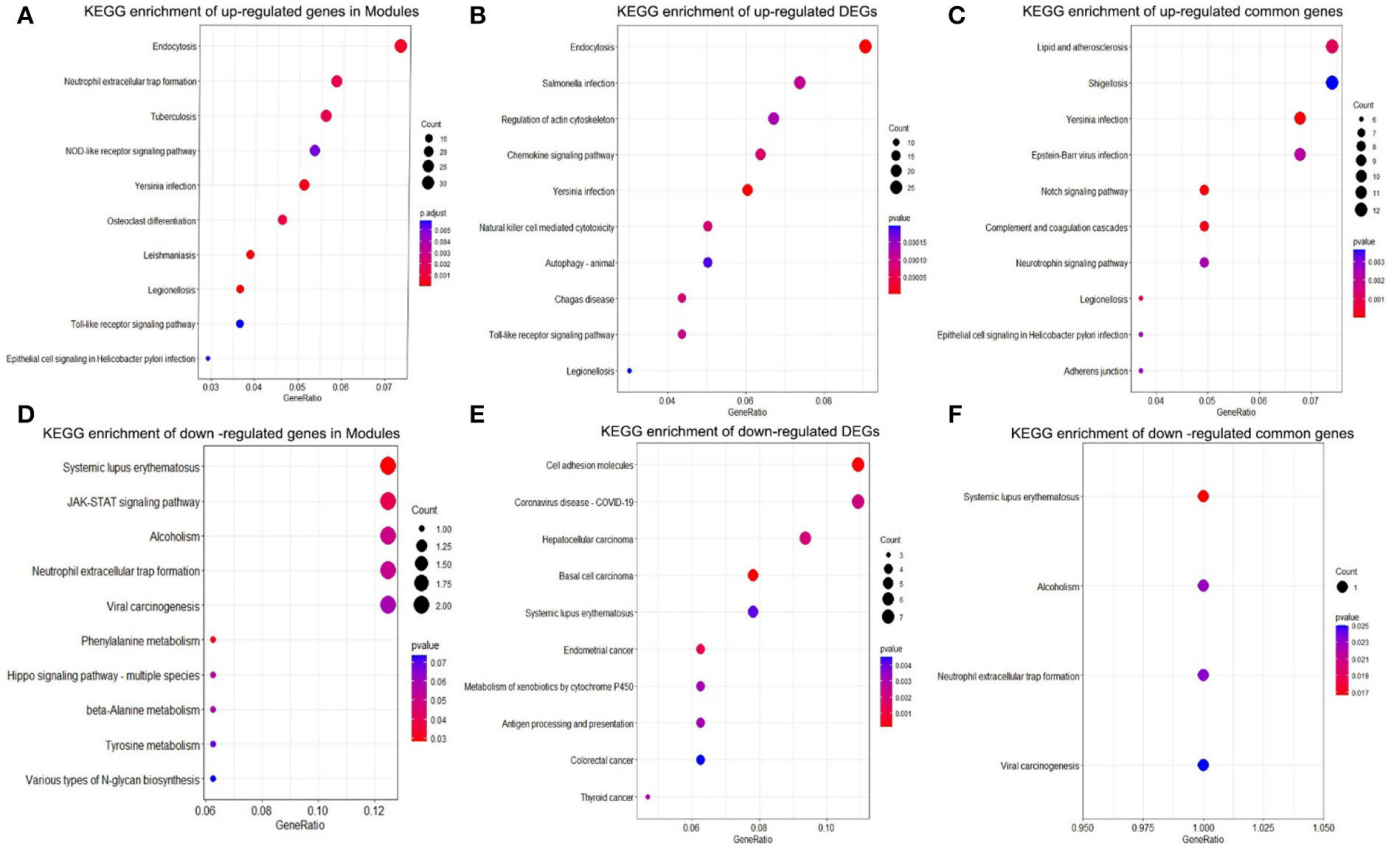
KEGG pathway enrichment analysis. **(A)** Top 10 KEGG pathway enrichment results of upregulated genes in the modules. **(B)** Top 10 KEGG pathway enrichment results of upregulated DEGs. **(C)** Top 10 KEGG pathway enrichment results of upregulated common genes. **(D)** Top 10 KEGG pathway enrichment results of downregulated genes in the modules. **(E)** Top 10 KEGG pathway enrichment results of downregulated DEGs. **(F)** KEGG pathway enrichment results in downregulated common genes. KEGG, Kyoto encyclopedia of genes and genomes; DEGs, differentially expressed gene.

**Figure 5:**
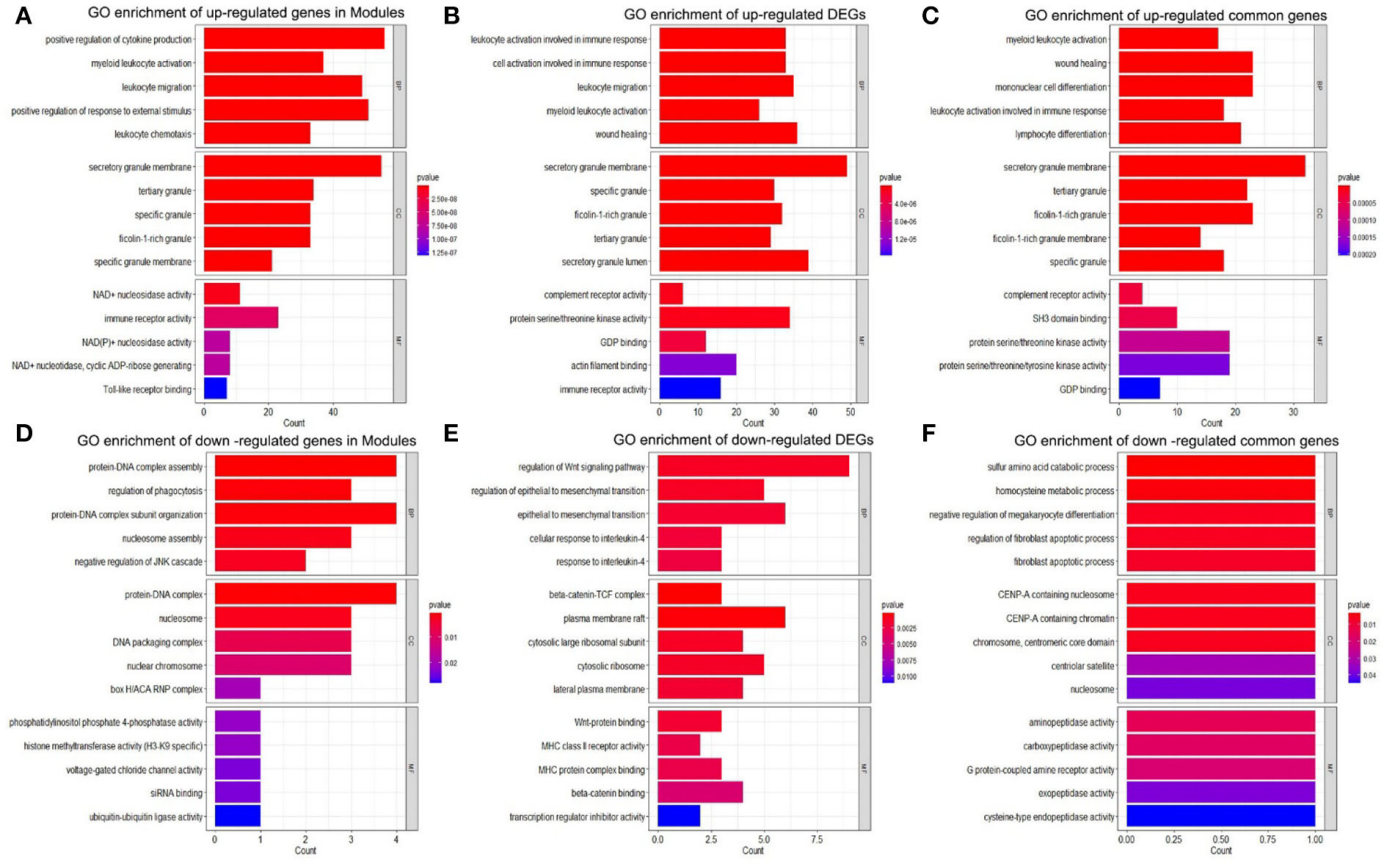
GO enrichment analysis. **(A)** GO enrichment of upregulated genes in the modules. **(B)** GO enrichment of upregulated DEGs. **(C)** GO enrichment of upregulated common genes. **(D)** GO enrichment of downregulated genes in Modules. **(E)** GO enrichment of downregulated DEGs. **(F)** GO enrichment of downregulated common genes. DEGs, differentially expressed genes; GO, gene ontology.

The KEGG pathway was analyzed to explore the biological function of upregulated genes and downregulated genes in DEGs, modules, and common genes. As shown in Figures 4A–C, the up regulated genes in DEGs, modules, and common genes were mainly enriched in Yersinia infection, Neutrophil extracellular trap formation, Epstein-Barr virus infection, complement and coagulation cascades, neurotrophin signaling pathway, legionellosis, and epithelial cell signaling in the *Helicobacter pylori* infection (Table 1). The downregulated genes in DEGs, modules, and common genes were mainly enriched in systemic lupus erythematosus, alcoholism, neutrophil extracellular trap formation, and viral carcinogenesis (Figures 4D–F; Supplementary Table S1). In addition, after the GO analysis of upregulated genes and downregulated genes in DEGs, modules, and common genes, it was found that these genes were mainly enriched in biological functions related to inflammation and regulation of immune response. The GO analysis of upregulated genes in DEGs, modules, and common genes was primarily enriched in myeloid leukocyte activation, leukocyte activation involved in immune response, lymphocyte differentiation, secretory granule membrane, tertiary granule, complement receptor activity, and immune receptor activity (Figures 5A–C; Table 2). The GO analysis of downregulated genes in DEGs, modules, and common genes was mainly concentrated in the cell growth process metabolic pathway and so on (Figures 5D–F; Supplementary Table S2).

**Table 1:** KEGG analysis of upregulated common genes in AUVP.

**ID**	**Description**	**Count**	***p*-value**
hsa04330	Notch signaling pathway	8	0.0000191
hsa05135	Yersinia infection	11	0.0000825
hsa04610	Complement and coagulation cascades	8	0.000267237
hsa05134	Legionellosis	6	0.000877613
hsa05417	Lipid and atherosclerosis	12	0.001148697
hsa05169	Epstein-Barr virus infection	11	0.002235012
hsa04722	Neurotrophin signaling pathway	8	0.002467031
hsa05120	Epithelial cell signaling in *Helicobacter pylori* infection	6	0.002566374
hsa04520	Adherens junction	6	0.002758126
hsa05131	Shigellosis	12	0.003666819

**Table 2:** GO analysis of upregulated common genes in AUVP.

**ID**	**Ontology**	**Description**	**Count**	***p*-value**
GO:0002274	BP	Myeloid leukocyte activation	17	2.07E-07
GO:0042060	BP	Wound healing	23	4.47E-07
GO:1903131	BP	Mononuclear cell differentiation	23	5.25E-07
GO:0002366	BP	Leukocyte activation involved in immune response	18	8.16E-07
GO:0030098	BP	Lymphocyte differentiation	21	9.60E-07
GO:0030667	CC	Secretory granule membrane	32	7.12E-17
GO:0070820	CC	Tertiary granule	22	2.29E-14
GO:0101002	CC	Ficolin-1-rich granule	23	3.08E-14
GO:0101003	CC	Ficolin-1-rich granule membrane	14	6.59E-13
GO:0042581	CC	Specific granule	18	1.10E-10
GO:0004875	MF	Complement receptor activity	4	3.29E-05
GO:0017124	MF	SH3 domain binding	10	4.45E-05
GO:0004674	MF	Protein serine/threonine kinase activity	19	0.000108993
GO:0004712	MF	Protein serine/threonine/tyrosine kinase activity	19	0.000174533
GO:0019003	MF	GDP binding	7	0.000204491

### Construction of PPI and screening hub genes identification

**Figure 6:**
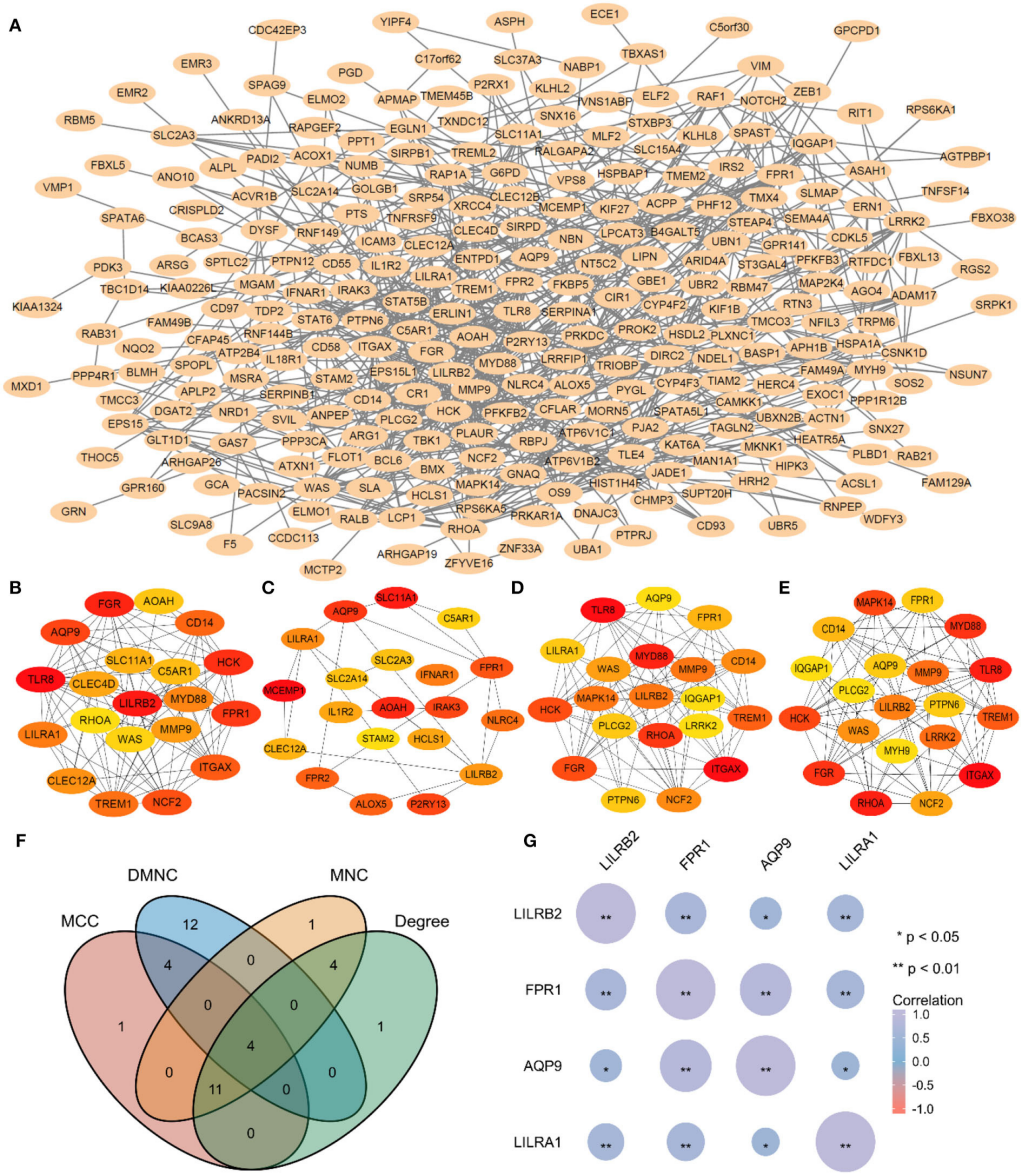
Hub genes identification. **(A)** Construction of PPI using 332 genes. **(B)** The Top 20 gene of MNC algorithm in PPI. **(C)** The Top 20 genes of DMNC algorithm in PPI. **(D)** The Top 20 genes of MCC algorithm in PPI. **(E)** The Top 20 genes of Degree algorithm in PPI. **(F)** The Venn plot of the intersection between four algorithms. **(G)** Pearson's correlation coefficient and correlation significance *p*-value among four hub genes of AUVP. MNC, maximum neighborhood component; DMNC, density of maximum neighborhood component; MCC, maximal clique centrality.

In this study, 332 key genes of AUVP were introduced into the online STRING database to construct the interaction between differential gene proteins. The PPI network between 332 genes had 156 nodes and 232 edges (Figure 6A). The CytoHubba plug-in of Cytoscape software was used to screen the hub genes among the 332 key genes of AUVP. According to the four scoring algorithms of MNC, DMNC, MCC and Degree of the CytoHubba plug-in, each gene in the PPI network was scored, and the genes were sorted according to the score of each algorithm. The first 20 genes are selected in each algorithm (Figures 6B–E), and the genes obtained by intersection are determined to be the hub genes of AUVP. Finally, four hub genes were obtained (Figure 6F). Pearson's correlation coefficient and correlation significance p-value among four hub genes are shown in Figure 6G.

### Hub genes identification

**Figure 7:**
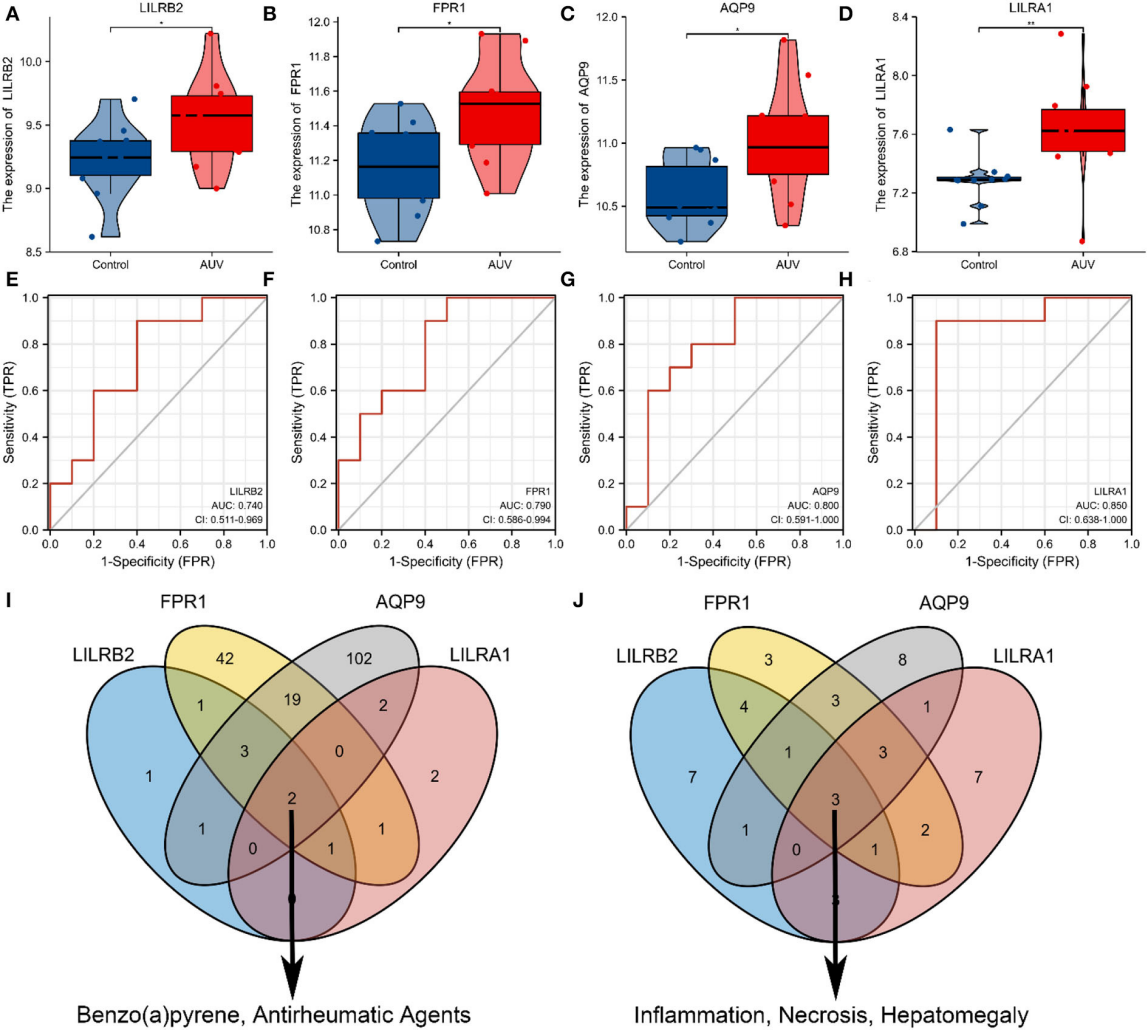
Exploring the functions of four hub genes. **(A)** Expression of LILRB2 mRNA among Normal and AUVP. **(B)** Expression of FPR1 mRNA among Normal and AUVP. **(C)** Expression of AQP9 mRNA among Normal and AUVP. **(D)** Expression of LILRA1 mRNA among Normal and AUVP. **(E)** ROC curve validated the sensitivity and specificity of LILRB2 as a predictive biomarker for AUVP prognosis. **(F)** ROC curve validated the sensitivity and specificity of FPR1 as a predictive biomarker for AUVP prognosis. **(G)** ROC curve validated the sensitivity and specificity of AQP9 as a predictive biomarker for AUVP prognosis. **(H)** ROC curve validated the sensitivity and specificity of LILRA1 as a predictive biomarker for AUVP prognosis. **(I)** The Venn plot of the four hub genes related to chemicals in the CTD. **(J)** The Venn plot of the four hub genes related to diseases in the CTD (*p < 0.05; **p < 0.01; ns, the difference is not statistically significant; CTD, Comparative Toxicogenomics Database).

The expression patterns of 4 hub genes between AUVP patients and normal samples demonstrated that LILRB2, FPR1, AQP9, and LILRA1 were increased in AUVP (*p* < 0.05) (Figures 7A–D). ROC curve analysis was performed with the “pROC” package to compare the diagnostic efficacy of four hub genes in patients with AUVP. LILRB2, FPR1, AQP9, and LILRA1 have sure accuracy in diagnosing AUVP (0.9 > AUC > 0.7, Figures 7E–H). More details of the four hub genes are listed in Table 3. Therefore, LILRB2, FPR1, AQP9, and LILRA1 can be used as hub genes in the pathogenesis of AUVP.

**Table 3:** The hub genes in Acute Unilateral Vestibulopathy.

**Gene**	GeneCards identifier*	**Full name of the gene**	Gene-related diseases*
LILRB2	GC19M066340	Leukocyte Immunoglobulin Like Receptor B2	Hymenolepiasis
FPR1	GC19M051745	Formyl Peptide Receptor 1	Susceptibility To Localized Juvenile Periodontitis; Periodontitis, Aggressive; Pulmonary Coin Lesion; Diamond-Blackfan Anemia 2 (DBA2); Aggressive Periodontitis.
AQP9	GC15P058138	Aquaporin 9	Hydrarthrosis; Polyhydramnios; Infective Endocarditis; Bullous Keratopathy; Constipation.
LILRA1	GC19P054593	Leukocyte Immunoglobulin Like Receptor A1	Immune System

* From the GeneCards database (www.genecards.org).

### Exploring the common functions of hub genes in the comparative toxicogenomics database

We can access this information by searching for target genes and looking up the chemicals and disease linked to the four hub genes in the CTD. The four hub genes were related to chemicals Benzo(a)pyrene and antirheumatic agents (Figure 7I). In addition, inflammation, necrosis, and hepatomegaly have strong links with the four hub genes in CTD (Figure 7J). This indicated that chemicals [benzo(a)pyrene and antirheumatic agents] and diseases (inflammation, necrosis, and hepatomegaly) play a significant role in the initiation and progression of the AUVP.

## Discussion

AUVP is the third inducing factor of secondary functional dizziness, second only to BPPV and vestibular migraine (1). Although many physiological and pathological mechanisms and etiological hypotheses of AUVP have been reported, the true etiology of AUVP is still unknown. Therefore, it is urgent and necessary to improve the understanding of the molecular mechanisms of AUVP and develop therapeutic strategies that aim at the targeting gene. First, the dataset of AUVP samples was analyzed by WGCNA R-package to obtain the relationship between the twelve gene expression modules and the clinical phenotype (group). It was found that the MEdarkturquoise module had the most considerable significant difference among all modules. Another method was used to perform the DEGs on the samples by the “limma” package, and 776 DEGs were screened. The common genes of the MEdarkturquoise module and 776 DEGs would be further analyzed. KEGG pathway analysis results suggest that the pathologies of AUVP may probably be associated with bacterial and viral infection pathways. The results of GO analysis indicated that AUVP might be related to the inflammatory response and regulation of immune response. Then constructed PPI network and four hub genes were identified by the Cytoscape software using four scoring algorithms of the CytoHubba plugin (MNC, DMNC, MCC, and Degree). It is reported that biological networks are heterogeneous, so it is reasonable to use more than one method of CytoHubba to screen the hub genes, and it was also found that the essential proteins filtered by the four algorithms of MNC, DMNC, MCC, and Degree have a better performance on the precision than the other methods (28). ROC curve analysis suggests that four hub genes have certain accuracy in diagnosing AUVP (0.9 > AUC > 0.7). Finally, the four hub genes of AUVP identified by the two methods were LILRB2, FPR1, AQP9, and LILRA1. Therefore, we believe that the four hub genes and inflammation play a vital role in the pathogenesis of AUVP. Next, the four hub genes will be used as the entry point to explore the role of inflammation in the pathogenesis of AUVP.

As for the viral infection of vestibular neuronitis, some researchers believe that viruses causing upper respiratory tract infection, such as influenza virus, adenovirus, herpes simplex virus (HSV), cytomegalovirus, Epstein Barr virus, and parainfluenza virus, are related to vestibular neuronitis because 43 and 46% of patients with vestibular neuronitis have shown previous or concurrent viral infection of the upper respiratory tract (29). HSV-1 is the most common cause of vestibular nerve and ganglion virus infection among them. The autopsy showed that HSV-1 DNA, CD8^+^T lymphocytes, cytokines, and chemokines were present in two-thirds of human vestibular ganglia (30), and injection of HSV-1 into a mouse model resulted in vestibular dysfunction of infected vestibular ganglion cells, such as vestibular neuritis (9, 30). Vestibular neuro virus infection or anterior vestibular artery ischemia is cause of vestibular neuritis. In addition, recent studies on the immune-mediated mechanism as the etiology of vestibular neuritis have been reported (9, 29, 30); the immune imbalance between T- helper cells and T- suppressor cells is associated with vestibular neuritis, similar to that observed in multiple sclerosis (9, 29). In addition, the functional enrichment analysis results of this study suggest that bacterial and viral infections are associated with AUVP development, including Epstein-Barr virus infection, Yersinia infection, legionellosis, and epithelial cell signaling in *Helicobacter pylori* infection. Inflammation and regulation of immune response also have been involved in the initiation and progression of AUVP. Therefore, the inflammation and immune response caused by virus infection may play an essential role in the pathogenesis of AUVP.

The previous study of the GSE146230 dataset has shown that the neutrophil-mediated immune pathway promotes AUVP development by mediating thrombotic changes and inflammation in vestibular organs with one bioinformatics method (18). In the current study, bioinformatic analyses were performed two times using two independent methods, and four hub genes were identified and validated. Among them, LILRA1 (CD86i, LIR6), a group I receptor that binds to HLA-C free heavy chains, has a lower affinity than LILRB1 and LILRB2 (31). The expression of LILRA1 is found on monocytes and macrophages. Anti-LILRA1 monoclonal antibody (clone m467) does not bind neutrophils (32). Additionally, LILRA1-specific peptides were not detectable in most proteomics studies of neutrophil-derived products. This indicates neutrophils expressed no or very little LILRA1 (33). LILRB2 (LIR2, CD85d, and ILT4), which contains four Ig-like domains, is a receptor for classical and non-classical HLA-I molecules (34–37). One study localized the expression of LILRB2 to particles. It showed that neutrophils were stimulated with fMLP, PS, or TNF α, resulting in upregulation of LILRB2 expression, which was accompanied by exocytosis of granules (38). The degranulation and phagocytosis of neutrophils were inhibited by cross-linking LILRB2 and HLA-G (38). During mid-and late-activation phases of the neutrophil lifecycle, LILRB2 modulates immune responses (33). FPR1 and its variants FPR like 1 (FPRL1) plays a critical role in cell proliferation, angiogenesis, and signaling pathways of neuroinflammation (39). In recent years, many pharmacological studies have demonstrated that FPR1 can effectively control neuroinflammation by inhibiting the production of various proinflammatory mediators, such as TNF-α and IL-1β, otherwise inducing IL-10 and IL-1RA expression (40). In the course of demyelination, FPR1 causes and maintains glial cell activation. Therefore, FPR1 is an essential component of innate immunity in chronic degenerative diseases such as multiple sclerosis (41). AQP9 seems responsible for neutrophil migration, as Aqp9-KO mice show reduced neutrophil migration to fMLP (42, 43). Patients with systemic inflammatory response syndrome (SIRS) show increased AQP9 expression in neutrophils compared to healthy controls (42, 44). It has been reported that AQP9 expression in astrocytes, ependymocytes, tanycytes, endothelial cells of pial vessels, and dopaminergic neurons of the midbrain in the CNS (45, 46). Although there were few research reports on the role of four hub genes in AUVP, as discussed above, we found that all four hub genes are related to inflammation in the CTD database. Therefore, the four hub genes are also related to neutrophils, modulate immune responses, and express on immune cells. They could be used as the entry point to explore the role of inflammation, immune responses, and Immune cells in the development of AUVP.

Furthermore, our research also has some limitations. The four hub genes screened in this study have not been verified by experiments, for the time being, we will use four hub genes as a breakthrough point to design a new topic and study the specific role of four hub genes in AUVP. Our analysis results are based on a small sample size, which may not provide sufficient evidence to support our hypothesis. More samples need to be further studied to determine the accuracy of hub genes in the diagnosis of AUVP and the relationship between hub genes level and future treatment patterns, and to determine the immune and inflammation-related pathological mechanisms behind AUVP.

## Conclusion

To sum up, the vestibular neurons are damaged following bacterial and viral infection. Then inflammation and immune responses are activated as well as immune proteins like LILRB2, FPR1, AQP9, and LILRA1 are produced in patients with AUVP to regulate their immune responses. However, the related mechanism still needs to be further explored. Although some reports suggest that viral infection is more critical in AUVP, we believe that bacterial infection may be equally important in the pathogenesis of AUVP. Therefore, the inflammation and immune response play a vital role in developing AUVP. Though it is still not clear about the specific position and mechanism of four hub genes in AUVP, the high expression levels suggest that they would be an essential target for AUVP diagnosis and treatment.

## Data availability statement

The original contributions presented in the study are included in the article/Supplementary material, further inquiries can be directed to the corresponding author.

## Author contributions

JH and YC designed the study. YC carried out the bioinformatic analysis and wrote the manuscript. JZ, YZ, CL, and BL reviewed and revised the manuscript. All authors read and approved the final manuscript.

## Conflict of interest

The authors declare that the research was conducted in the absence of any commercial or financial relationships that could be construed as a potential conflict of interest.

## Publisher's note

All claims expressed in this article are solely those of the authors and do not necessarily represent those of their affiliated organizations, or those of the publisher, the editors and the reviewers. Any product that may be evaluated in this article, or claim that may be made by its manufacturer, is not guaranteed or endorsed by the publisher.
