# Supplementary material for: Identification of hub genes and pathophysiological mechanism related to acute unilateral vestibulopathy by integrated bioinformatics analysis
Source: Front Neurol. 2022 Sep 27;13:987076. doi: 10.3389/fneur.2022.987076 (PMC9552803; doi:10.3389/fneur.2022.987076)
Supplement: Supplementary file 1 [file Table_1.DOCX]

**Table S1.** KEGG analysis of down-regulated common genes in AUVP.

| ID | Description | Count | p-value |
| --- | --- | --- | --- |
| Hsa05322 | Systemic lupus erythematosus | 1 | 0.016668709 |
| hsa05034 | Alcoholism | 1 | 0.022919475 |
| Hsa04613 | Neutrophil extracellular trap formation | 1 | 0.023287168 |
| hsa05203 | Viral carcinogenesis | 1 | 0.025003064 |
