# Supplementary material for: Identification of hub genes and pathophysiological mechanism related to acute unilateral vestibulopathy by integrated bioinformatics analysis
Source: Front Neurol. 2022 Sep 27;13:987076. doi: 10.3389/fneur.2022.987076 (PMC9552803; doi:10.3389/fneur.2022.987076)
Supplement: Supplementary file 2 [file Table_2.DOCX]

**Table S2.** GO analysis of down-regulated common genes in AUVP.

| ID | Ontology | Description | Count | p-value |
| --- | --- | --- | --- | --- |
| GO:0000098 | BP | sulfur amino acid catabolic process | 1 | 0.003187672 |
| GO:0050667 | BP | homocysteine metabolic process | 1 | 0.00414232 |
| GO:0045653 | BP | negative regulation of megakaryocyte differentiation | 1 | 0.005731708 |
| GO:2000269 | BP | regulation of fibroblast apoptotic process | 1 | 0.006049332 |
| GO:0044346 | BP | fibroblast apoptotic process | 1 | 0.007001696 |
| GO:0043505 | CC | CENP-A containing nucleosome | 1 | 0.005499948 |
| GO:0061638 | CC | CENP-A containing chromatin | 1 | 0.005499948 |
| GO:0034506 | CC | chromosome, centromeric core domain | 1 | 0.00580476 |
| GO:0034451 | CC | centriolar satellite | 1 | 0.03232513 |
| GO:0000786 | CC | nucleosome | 1 | 0.038862278 |
| GO:0004177 | MF | aminopeptidase activity | 1 | 0.014737375 |
| GO:0004180 | MF | carboxypeptidase activity | 1 | 0.016613248 |
| GO:0008227 | MF | G protein-coupled amine receptor activity | 1 | 0.019234327 |
| GO:0008238 | MF | exopeptidase activity | 1 | 0.038150672 |
| GO:0004197 | MF | cysteine-type endopeptidase activity | 1 | 0.044751932 |
